# A Cross-Sectional Study of the Impact of the COVID-19 Pandemic on Previously Diagnosed Patients With Fibromyalgia

**DOI:** 10.7759/cureus.29337

**Published:** 2022-09-19

**Authors:** Vikas Saxena, Kiran Jakhar, Satendra Kumar, Pihu Sethi, Hariom K Solanki

**Affiliations:** 1 Department of Orthopedics, Government Institute of Medical Sciences, Greater Noida, IND; 2 Department of Psychiatry, Government Institute of Medical Sciences, Greater Noida, IND; 3 Department of Surgery, Government Institute of Medical Sciences, Greater Noida, IND; 4 Department of Dermatology, Venereology & Leprosy, Government Institute of Medical Sciences, Greater Noida, IND; 5 Department of Community Medicine, Government Institute of Medical Sciences, Greater Noida, IND

**Keywords:** sars-cov-2, impact, revised fibromyalgia impact questionnaire (fiqr), covid-19, fibromyalgia

## Abstract

Introduction

Coronavirus disease 2019 (COVID-19) was a one-of-its-kind pandemic due to its high infectivity and mortality rate. Prolonged lockdown periods imposed during the pandemic saved many lives but, on the other hand, had a huge psychological and clinical impact on patients suffering from chronic medical illnesses.

Aims

This study aimed to find the impact of the COVID-19 pandemic on patients with previously diagnosed fibromyalgia.

Methods

A prospective observational study including the cohort of previously diagnosed cases of fibromyalgia as per American College of Rheumatology (ACR) 2010 criteria where the patients were evaluated by an online survey for socio-demographic profile, subjective improvement, and objective improvement in quality of life by the Fibromyalgia Impact Questionnaire-Revised (FIQR). Pre and post-COVID-19 scores were analyzed. The statistical procedure used included the chi-square test.

Results

A total of 78 subjects were recruited for the study, with a female preponderance (75%) and mean (SD) age of 37.2 (9.2) years. The duration of symptoms was nine to 12 months followed by more than 12 months at the time of the first consultation for the majority of subjects. Sixty-five percent (65%) of subjects had no improvement or deterioration on FIQR.

Statistical analysis

This included mean, standard deviation, proportions, percentages, and the chi-square test.

Conclusion

COVID-19 had a significant negative impact on patients with fibromyalgia even on continued pharmacological treatment. However, there was no statistically significant data on the comparison of the overall mean score of FIQR and each domain individually with the continuation of treatment and improvement of symptoms.

## Introduction

Fibromyalgia is a chronic, centralized pain sensitivity disorder involving the musculoskeletal system with a widespread and fluctuating course of pain episodes accompanied by psychological as well as somatic symptoms [1]. Basically, it increases the processing of pain in the central nervous system [2] with a mean population prevalence of 2.7% worldwide [3]. The most affected population is women in the age group of 30-55 years [4]. The pathogenesis of fibromyalgia is multi-factorial and infections [5] and psychological and physical stressors tend to aggravate the illness. A study by Barnes Marroquín et al. (2022) emphasized the fact that there was a significant deterioration of fibromyalgia symptoms during the COVID-19 pandemic, which included pain, anxiety, and depression on the Fibromyalgia Impact Questionnaire and other specific questionnaires (the Patient Health Questionnaire and the Generalized Anxiety Disorder questionnaire) [6].

 A study by Aloush et al. (2021) found high levels of pain, anxiety, depression, sleep disturbances, and subjective perception of worsening among FM patients during the COVID-19 outbreak and lockdown measures. Difficulties entailed by social distancing, economic issues, difficulties in accessing medical facilities, and complimentary treatment led to the deterioration [7].

The novel coronavirus disease 2019 (COVID-19) pandemic caused by severe acute respiratory syndrome coronavirus-2 (SARS-COV-2) led to a government-imposed lockdown worldwide to reduce the virus contamination rate and limit hospital overcrowding [8]. This lockdown was the mainstay strategy to control the infection either by isolation or by quarantine but it significantly impacts the daily lives of the general population. There was a significant impact on the psychological well-being and emotional health of the individual, as there was limited access to medical and complementary treatment during periods of lockdown and difficulty acquiring necessary medications [7,9].

With the multifactorial etiology of fibromyalgia, infections, stressors, autoimmune causation, COVID-19 causing a flu-like infection, and stressful environments, the imposition of a lockdown would have impacted the lifestyle of participants in terms of difficulty in procuring medicines and physiotherapy. The current study aimed to evaluate the impact of the lockdown during COVID-19 in previously diagnosed patients with fibromyalgia and to assess any socio-demographic parameters if correlated.

## Materials and methods

This was an observational prospective study conducted on a cohort of participants previously diagnosed with fibromyalgia at a tertiary care center in Western Uttar Pradesh. After ethical approval by the Institutional Ethical Committee and following ethical procedures, fibromyalgia participants were recruited for the study. The sample for the original study was 126 participants previously diagnosed with fibromyalgia by the American College of Rheumatology (ACR) 2010 criteria between March 2019 and September 2019 [10].

The participants were contacted through an online survey. Those participants who were willing to participate in the study and agreed to give informed consent were recruited. All the participants were asked to give written consent through online forms and for those who didn’t have access to technology; verbal consent was taken through telemedicine. At the start of the study, the participants were informed briefly about the purpose of the study. Participants were evaluated using an online survey that included a semi-structured proforma designed to collect information regarding socio-demographic variables and Fibromyalgia Impact Questionnaire-Revised (FIQR).

The FIQR has three domains - function, overall impact, and symptoms, and the individual domain duration was for the past seven days. The answers were given on a Likert scale of 0-10. The function domain had nine questions, and their answers range from no difficulty to very difficult. The overall domain had two questions and their answers range from never to always. The symptoms domain had 10 questions and the answers range from no to very much. For scoring, the scores for each of the three domains (function, overall, and symptoms) were summed up, and then the domain 1 score was divided by 3, domain 2 scores by 1 (that is, it is unchanged), and domain score 3 by 2. Finally, the three resulting domain scores were added to obtain the total FIQR score.

The online survey was collected within three months of the initiation of the study. Data analysis was done using appropriate statistical software. Frequency distribution in terms of mean and standard deviation, proportions, and percentage was carried out for socio-demographic details. The statistical procedure used included the chi-square test.

## Results

A total of 78 participants who were previously diagnosed with fibromyalgia by the ACR 2010 criteria and took the online survey were recruited for the study [11]. They had: 1. Widespread pain index (WPI) ≥ 7 and symptom severity scale (SSS) score ≥ 5 OR WPI of 3-6 and SSS score ≥ 9; 2. Symptoms had been present at a similar level for at least three months; 3. The patient did not have a disorder that would otherwise explain the pain.

Out of the 78 participants, the majority were female accounting for approximately three-quarters (¾) of the sample size (75.64%). The mean age of participants was 37.2 years. The majority of participants were literate and were educated up to graduation or equivalent and housewife or skilled worker (Table 1).

**Table 1:** Socio-demographic variables of fibromyalgia subjects

S. No.	Parameters	Number	Percentage
1.	Gender		
	Male	19	24.35%
	Female	59	75.64%
2.	Age distribution		
	<20 Years	0	0%
	20-40 Years	57	73.07%
	40-60 Years	19	24.35%
	>60 Years	02	02.56%
3.	Education		
	Illiterate	08	10.25%
	Upto High School	23	29.48%
	Bachelor or Equivalent	36	46.15%
	Master or Equivalent	11	14.10%
4.	Employment		
	Employed	29	37.17%
	Unemployed	04	05.12%
	Student	04	05.12%
	Housewife	41	52.64%

The duration of symptoms at the time of the first consultation for the majority of participants was lasting for nine to 12 months followed by more than 12 months. While at the time of the index study, the majority of participants took treatment for six to nine months after the diagnosis of fibromyalgia (Table 2).

**Table 2:** Duration of symptoms of subjects in the study group at the time of the first consultation and at the time of the study

S. No.	Duration of symptoms	Number of subjects at the time of the first consultation (N=78)	Number of subjects at the time of the study (N=78)
1.	3-6 months	16	9
2.	6-9 months	13	35
3.	9-12 months	27	27
4.	>12 months	22	7

Twenty-one participants (26.9%) had stopped treatment for fibromyalgia at the time of the teleconsultation. Among these 21 participants, seven participants were free of symptoms so they discontinued while the other 14 participants stopped treatment due to financial constraints or confinement due to the COVID-19 pandemic (Table 3). There was no stoppage of fibromyalgia medication due to non-availability, as the government made no restrictions on drug stores.

**Table 3:** Impact of COVID-19 on the previously diagnosed case of fibromyalgia in terms of subjective assessment and objective assessment using FIQR FIQR: Fibromyalgia Impact Questionnaire-Revised

Assessment	The group as per continuation of treatment (N= 78)	Cured	Improved	Same	Deteriorated
Subjective Assessment	Treatment continued	7	49	1	0
	Treatment Discontinued	7	3	4	7
Objective Assessment	Treatment continued	0	20	30	7
	Treatment Discontinued	0	6	6	9

**Figure 1:**
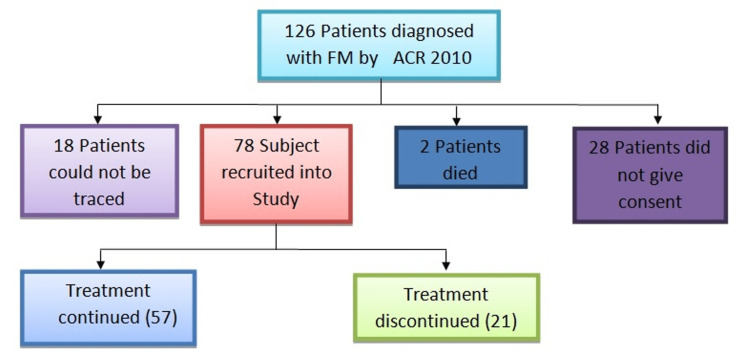
Flowchart 1

**Figure 2:**
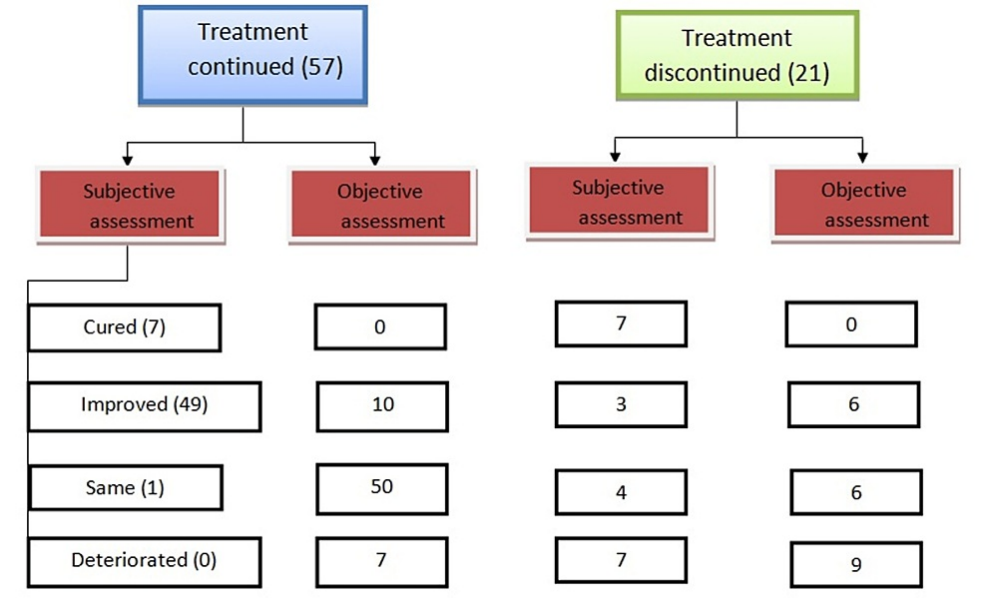
Flowchart 2

Among 57 participants who continued treatment, 56 participants had subjective improvement on the symptom subscale of FIQR while one participant had no improvement in symptoms. Out of 21 participants who stopped treatment, 11 participants had no improvement or deterioration on subjective parameters (Figure 1, Figure 2).

In the comprehensive objective assessment by FIQR, 37 participants showed deterioration or no improvement while 20 participants had objective improvement in the continued treatment group (Table 4).

**Table 4:** Association of selected variables with changes in mean score over the COVID-19 pandemic

		Change in Score		P-value (t-test)
	Continued treatment	Mean	SD	
Overall for all domains	No (21)	1.47	17.28	0.289
	Yes (57)	5.87	15.79	
Domain 1 score	No (21)	49.90	5.50	0.915
	Yes (57)	49.77	4.59	
Domain 2 score	No (21)	11.14	1.74	0.345
	Yes (57)	11.49	1.31	
Domain 3 score	No (21)	53.09	4.66	0.038
	Yes (57)	55.35	4.02	
Gender	Male (19)	12.49	16.49	0.015
	Female (59)	2.17	15.43	

On analyzing the overall mean score of FIQR and each domain individually, there was no statistical difference in any of the domains in relation to continued treatment and symptom improvement.

## Discussion

As per the literature available, stressors either cause, precipitate, or aggravate the symptoms of fibromyalgia. The current COVID-19 pandemic not only causes the symptoms of infection but, in a real sense, provides a gateway to a multitude of various psychological stressors and mental health issues [12]. The current study is meant to objectively assess the impact of COVID-19 on fibromyalgia symptoms.

In the current study, the majority of participants were female (approximately 75%) and in the age group of 20-40 years, which correlates with the data on fibromyalgia, as this illness has a female preponderance [7,8,13-15]; however, the age group in the index study is a near-decade younger in contrast to various studies [7,13-15]. Nearly half of the participants were graduates or equivalent (college-going) in the current study, similar to the study by Aloush et al. (2021) [7]. Whereas in the other studies, the majority of the population was educated up to primary school or high school [13,14]. More than half of the participants were women who were housewives followed by the employed/skilled population (37%), similar to the study by Cankurtaran et al. (2021) [13].

In the current study, the majority of participants had a duration of illness of nine to 12 months followed by more than 12 months, whereas, in other studies, the duration of illness varied from one to six years [9,14]. Approximately one-fourth of the participants discontinued the treatment during the pandemic, which is in consonance with various studies where pandemics had led to treatment discontinuation in the form of medicines, contemporary treatment, exercise, or psychological intervention [7,9].

Among 57 participants who continued treatment, approximately 65% of participants had no improvement or deterioration on the comprehensive objective assessment by FIQR, which means an aggregative score of function, overall impact, and symptoms for the last seven days, which is in consonance with other studies [9,15]. The reason for this is that even despite pharmacological treatment, there are other modalities of treatment that were hampered during the lockdown, and COVID-19 itself had psychological effects on the general population. The patients suffering from fibromyalgia are already predisposed to psychological symptoms like fatigue and sleep disturbance, which form the core criteria of fibromyalgia.

There was an unexpected finding of subjective improvement in symptom reporting for some participants. The reason could be that during the lockdown period, family members had more time to spend together and care for each other. The functional output required during this period was also less, as no official work was expected from them. They were not allowed to go outdoors and stayed at home, which resulted in less fatigue and hence less reporting of symptoms.

No statistically significant difference was found when the mean score of FIQR and each domain individually was taken into account in relation to continued treatment and symptom improvement.

The current study is one of its kind to assess the impact of the COVID-19 lockdown on fibromyalgia. It is also one of its kind in India as a preliminary work but it emphasizes that the lockdown period had a deleterious effect on these patients and they need extra psychological care. However, there are certain limitations of the study. As this is a cross-sectional study, no causal relationship between COVID-19 imposed lockdown and deterioration of symptoms could be established. There is no objective assessment of the treatment response of participants prior to lockdown which would have provided the actual measure of deterioration due to COVID-19. The sample size of the study is also comparatively small.

## Conclusions

The current study reemphasized the fact that fibromyalgia is an illness predominately affecting females in the age group of 20-40 years. Fibromyalgia is an illness that has multifactorial associations, and one of its important factors is psychological stress. The COVID-19 pandemic is one of its kind, which has taken a huge psychological toll; hence, this study actually strengthens the fact that the COVID-19 imposed lockdown lead to a decline in the health status of patients, as it affected the treatment-seeking behavior. Also, the individuals who continued treatment had deterioration or no significant improvement in their symptoms. However, one minor exception to this finding is that a proportion of individuals reported subjective improvement, which was statistically insignificant. However, no prior objective assessment of the treatment response of participants was done, which would have provided actual deterioration. As the sample size of the study was also comparatively small, further research with a larger sample size to elucidate the explanation of these observations is warranted.
